# Influence of Stent Parameters and Post-Stenting Venographic Findings on Mid-Term Patency After Thrombectomy in May–Thurner Syndrome

**DOI:** 10.3390/diagnostics16071058

**Published:** 2026-04-01

**Authors:** Chang Hoon Oh, In Chul Nam, Doo Ri Kim, Jeong Sub Lee, Jeong Jae Kim, Hyoung Nam Lee, Sung-Joon Park, Youngjong Cho

**Affiliations:** 1 Department of Radiology, Samsung Medical Center, School of Medicine, Sungkyunkwan University, Seoul 06351, Republic of Korea; 2 Department of Radiology, Jeju National University Hospital, School of Medicine, Jeju National University, Jeju 63241, Republic of Korea; 3 Department of Radiology, Cheonan Hospital, College of Medicine, Soonchunhyang University, Cheonan 31151, Republic of Korea; 4 Department of Radiology, Korea University Ansan Hospital, College of Medicine, Korea University, Ansan 15355, Republic of Korea; 5 Department of Radiology, Gangneung Asan Hospital, College of Medicine, University of Ulsan, Gangneung 25440, Republic of Korea

**Keywords:** May–Thurner syndrome, deep vein thrombosis, iliac vein stenting, residual stenosis, venous patency, thrombectomy, endovascular treatment

## Abstract

**Background/Objectives**: In May–Thurner syndrome (MTS)-related iliofemoral deep vein thrombosis (DVT), stent failure remains an important concern; however, the prognostic value of stent parameters and immediate post-stenting venographic findings is not fully defined. This study aimed to evaluate the stent patency outcomes and analyze the effect of various stent-related parameters on the risk of stent occlusion. **Methods**: This retrospective study included 50 patients with acute iliofemoral DVT secondary to MTS, who underwent thrombectomy followed by iliac vein stenting. Stent diameter, oversizing, stent type, and post-stenting stenosis were evaluated as predictors of primary patency. Kaplan–Meier and Cox proportional hazards analyses were performed. **Results**: Nine (18%) patients developed stent occlusion within the first year. The overall primary patency rates were 88% and 81.2% at 1 and 2 years, respectively. Stent diameter, oversizing, and stent type were not significantly associated with patency. In contrast, residual stenosis ≥50% on completion venography was strongly predictive of occlusion (hazard ratio, 4.625; *p* < 0.001). Patients without significant residual stenosis demonstrated superior patency. **Conclusions**: Thrombectomy with stenting provides excellent mid-term patency in MTS-related DVT, and early stent failure occurs. Residual stenosis ≥50% after stent deployment is the only significant determinant of reocclusion, whereas stent diameter, oversizing, and stent type do not influence outcomes. Achieving complete luminal restoration during the index procedure is critical for achieving long-term patency.

## 1. Introduction

Deep vein thrombosis (DVT) of the iliofemoral veins can lead to serious complications such as pulmonary embolism and post-thrombotic syndrome (PTS). Left iliofemoral DVT is often associated with May–Thurner syndrome (MTS), an anatomic compression of the left common iliac vein by the overlying right common iliac artery [1,2]. MTS accounts for 18–49% of left lower-extremity DVT cases [3]. Chronic pulsatile arterial compression causes intraluminal fibrotic spurs and venous stenosis, which predispose to thrombosis [1]. Standard anticoagulation alone does not address the underlying venous outflow obstruction; patients with persistent iliac vein lesions or residual thrombus have a high risk of recurrent DVT and PTS [3,4].

Endovascular treatment has become the preferred approach for symptomatic MTS, particularly when complicated with acute DVT [4]. Typically, this involves catheter-directed thrombolysis or thrombectomy, followed by angioplasty and stent placement [5,6,7,8,9,10,11,12,13]. Stenting in patients with MTS is safe and effective, with primary patency rates on the order of 80–95% at mid- to long-term follow-up [4,5,6,7,8,9]. As a result, the current guidelines of vascular societies recommend iliac vein stenting in patients with significant iliac vein compression lesions, particularly when treating iliofemoral DVT [14].

Despite the excellent outcomes, stent failure (occlusion or significant in-stent restenosis) can occur in a subset of patients. Factors contributing to stent occlusion in MTS-related DVT are not fully understood. Therefore, this study aimed to evaluate the stent patency outcomes and analyze the effect of various stent-related parameters on the risk of stent occlusion.

## 2. Materials and Methods

The study was approved by the Institutional Review Board of our hospital (JEJUJNUH 2025-11-026), and the requirement for informed consent was waived owing to the retrospective nature of the study.

### 2.1. Study Population

**Figure 1:**
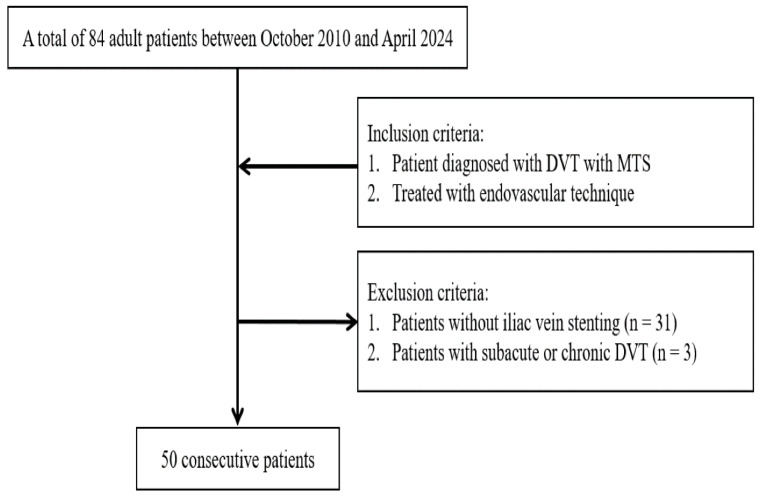
Case accrual process.

We retrospectively reviewed patients with acute iliofemoral DVT associated with MTS who underwent endovascular stenting of the iliac vein at our institution. Between October 2010 and April 2024, we identified 84 consecutive adult patients (aged ≥18 years) diagnosed with iliofemoral DVT secondary to MTS, who underwent thrombectomy. We excluded 31 patients who did not undergo stenting after thrombectomy and three patients with subacute DVT. Therefore, 50 patients were included in this study. Acute DVT was defined as symptom onset within 2 weeks and confirmed thrombosis in the iliac or common femoral veins on imaging. MTS is diagnosed based on imaging evidence of left common iliac vein compression by the right common iliac artery, either on venography or computed tomography, often manifested by focal stenosis and collateral veins in the iliac region. In all cases, the left iliac vein lesion was treated with stent placement after acute thrombus management. The indication for intervention was iliofemoral DVT with significant symptoms (limb swelling and pain) and the intent to prevent PTS. Figure 1 illustrates the accrual process.

### 2.2. Intervention Techniques

All procedures were performed by one of four interventional radiologists with 6, 8, 13, or 22 years of experience in venous interventions. The procedures were performed using an angiography suite (AlluraClarity FD20, Philips Healthcare, Best, The Netherlands; Artis Zee Ceiling, Siemens Healthcare, Erlangen, Germany).

First, local anesthesia (10–15 mL of 2% lidocaine) was injected using a 22G needle along the entrance point around the left popliteal vein under ultrasound (US) guidance and analgesia in the prone position. Venous access was achieved via the ipsilateral popliteal veins. Initially, a 14Fr sheath was inserted, followed by the passage of a 0.035″ hydrophilic wire into the inferior vena cava using a 5Fr catheter. Subsequently, a mixture of iodine contrast and normal saline solution was carefully injected while gradually withdrawing the 5Fr catheter to evaluate the extent and burden of the thrombus in the iliofemoral vein and to assess the location and severity of the iliac vein obstruction. Mechanical thrombectomy was performed as the primary treatment in all enrolled patients. For thrombectomy, the hub of an 11Fr braided sheath was trimmed to enable connection to a syringe, after which multiple aspiration thrombectomies were performed to remove the majority of the thrombus. Alternatively, an Angiojet (Boston Scientific, Marlborough, MA, United States of America [USA]) catheter was employed for single-session pharmacomechanical thrombectomy. Subsequently, an 8–14 mm-diameter balloon catheter was exchanged, and angioplasty was performed by inflating for 2–3 min at the usual 4–10 atmospheres. Following thrombus clearance, a self-expanding metallic stent was deployed across the left common iliac vein to relieve compression. The decision to perform stent placement was based primarily on venographic evidence of persistent iliac vein compression or significant residual stenosis after thrombectomy, rather than patient-related factors such as age. If venography demonstrated satisfactory venous outflow without significant residual obstruction after thrombectomy, additional stent placement was not performed. Stent selection (type and diameter) was performed at the operator’s discretion. Both venous-dedicated stents, specifically the Venovo venous stent (BD Peripheral Intervention, Tempe, AZ, USA) and non-dedicated stents (various commercially available vascular stents), were used in this study. Stent diameters ranged from 10 to 18 mm and were chosen based on the vessel size and extent of compression. As the diameter of the compressed segment could not be reliably measured, sizing was guided by the adjacent venous segment (such as the left external iliac vein). After stent deployment, a balloon angioplasty was performed to expand and appose the stent to the venous wall fully. Complete venography was performed to assess the results.

### 2.3. Data Collection

**Figure 2:**
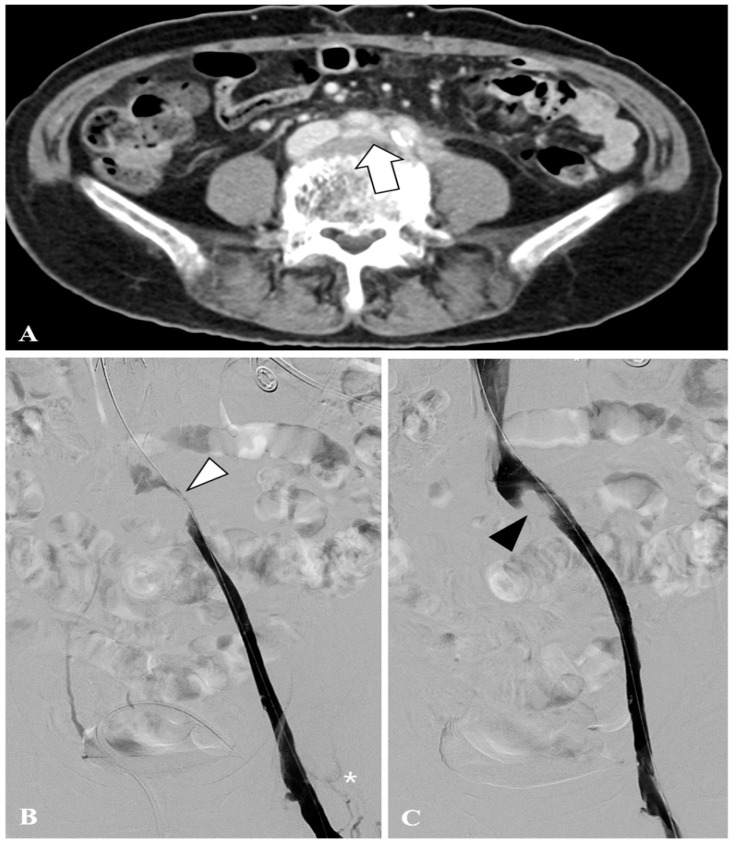
Endovascular treatment of May–Thurner syndrome-related iliofemoral deep vein thrombosis in a 66-year-old woman. (**A**) Contrast-enhanced computed tomography showing compression of the left common iliac vein (by the overlying right common iliac artery and L4 lumbar vertebral body (white arrow). (**B**) Conventional venography after thrombectomy demonstrates a severe stenosis (white arrowhead), along with collateral venous flow (asterisk). (**C**) Post-stenting venography shows partial relief of the stenosis; however, a residual luminal narrowing greater than 50% persists (black arrowhead). The collateral vein has resolved. This patient demonstrated stent reocclusion at the 1-month follow-up.

Age and sex data were also collected. The intervals between symptom onset and treatment were also assessed. We evaluated the following stent-related parameters as potential predictors of patency outcomes: **(a)** stent diameter, categorized into two groups: small (10 mm or 12 mm stents) and large (≥14 mm stents) diameters; **(b)** stent oversizing, assessed by comparing the stent diameter with the adjacent external iliac vein diameter, and categorized as ≥20% oversizing vs. <20%; **(c)** stent type, use of a vein-dedicate stent vs. a standard self-expanding stent (non-dedicated); **(d)** residual stenosis after stenting, defined as residual luminal narrowing ≥50% at the treated segment on venography (yes vs. no). This was assessed by comparing the diameter of the stenotic segment to the adjacent normal vein or the expected normal caliber; ≥50% diameter reduction was considered hemodynamically significant, and <50% diameter loss was considered non-significant stenosis. Intravascular ultrasound (IVUS) was not routinely used in this study; **(e)** residual collaterals, whether venous collateral channels remained visibly opacified on the post-stent venogram (yes vs. no), suggesting continued venous bypass of the iliac segment. Figure 2 shows representative venographic findings demonstrating the characteristic features of MTS.

### 2.4. Follow-Up

After hospital discharge, the patients received therapeutic-dose anticoagulation for the treatment of DVT. During the early study period, anticoagulation consisted primarily of low-molecular-weight heparin, which was subsequently switched to daily oral warfarin with a target INR of 2.0–3.0 for a minimum duration of 6 months. In the later years of the study period, direct oral anticoagulants were increasingly used according to evolving institutional practice. They underwent periodic follow-up to assess stent patency and clinical improvement. Follow-up evaluations included duplex ultrasonography at regular intervals (for example, 1 month, 3 months, 6 months, and then every 6–12 months) or venographic or computed tomography (CT) venographic studies, as indicated by the symptoms. Stent patency was defined as uninterrupted flow through the stented segment on imaging with no reocclusion. Stent occlusion was diagnosed if complete rethrombosis of the stented vein segment on imaging was observed or if re-intervention (thrombolysis or thrombectomy) was required due to rethrombosis. The timing of any occlusions was recorded. For patients without stent occlusion, the data were censored at the time of the last follow-up imaging to demonstrate patency.

### 2.5. Statistical Analysis

We used Kaplan–Meier analysis to estimate primary patency over time and compare patency across subgroups. A Kaplan–Meier survival curve for freedom from stent occlusion was generated, and log-rank tests were performed to compare patency between groups I and II based on stent diameter. In addition to diameter-based groups, we compared patency according to other categorical variables, including stent type (dedicated vs. non-dedicated), stent oversizing (≥20% oversizing vs. <20%), and post-stenting residual stenosis (<50% vs. ≥50%), using log-rank tests. Cox proportional hazards regression analysis was performed to identify factors associated with the risk of stent occlusion. Hazard ratios (HR), 95% confidence intervals (CI), and *p*-value were calculated for each variable. An HR > 1 indicates a higher risk of occlusion, and <1 indicates a protective effect. Statistical significance was set at *p* < 0.05. All statistical analyses were performed using IBM Statistical Package for the Social Sciences Statistics for Windows version 29 (IBM Corp., Armonk, NY, USA).

## 3. Results

Finally, we included 50 patients diagnosed with symptomatic MTS and acute DVT who underwent thrombectomy with iliac vein stenting. The study patients comprised 10 (20%) men and 40 (80%) women (mean age, 68 ± 15.7 years). Stents were successfully deployed in the left common iliac vein region in 100% of the patients. The stents used included dedicated venous stents (*n* = 13) and non-dedicated stents (*n* = 37). Group 1 (small diameter 10–12 mm) comprised 20 patients, and Group 2 (large ≥ 14 mm) comprised 30 patients. The choice of stent size generally correlated with the treating physician’s assessment of the vein size and degree of compression. Table 1 summarizes patient demographics.

In total, nine patients (18%) experienced reocclusion, including five (10%) in Group I and four (8%) in Group II. In the vein-dedicated stent group, stent occlusion occurred in one patient (2%), whereas eight patients (8%) in the non-vein-dedicated stent group experienced occlusion. On immediate post-stenting venography, a residual stenosis was observed in 13 patients (26%), including mild stenosis (<50%) in eight patients (16%) and severe stenosis (≥50%) in five patients (10%). Among those with severe stenosis, all five patients experienced reocclusion, whereas only one patient with mild stenosis developed occlusion. In patients without stenosis (*n* = 37), three experienced occlusion. No residual venous collaterals were observed on postprocedural venography. No major procedural complications (such as iliac vein rupture, pulmonary embolism during the intervention, or access site complications) were observed. Table 2 summarizes the results of the univariate analysis.

**Figure 3:**
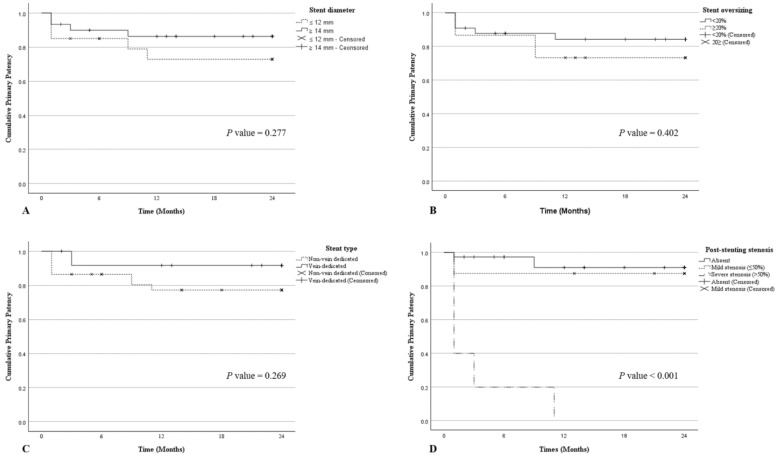
Kaplan–Meier curve showing 24-month cumulative primary patency stratified by venographic and procedural factors. (**A**) Stent diameter (≤12 mm vs. ≥14 mm). (**B**) Stent oversizing (≥20% vs. <20%). (**C**) Stent type (vein-dedicated vs. non-venous-dedicated). (**D**) Residual stenosis on post-stenting venography (absent, <50%, ≥50%).

The overall primary patency rates were 88% at 1 year and 81.2% at 2 years. Kaplan–Meier survival analysis was performed to assess cumulative primary patency rates according to stent parameters and post-stenting venographic findings. The Kaplan–Meier analysis revealed no statistically significant differences in primary patency according to stent diameter, stent oversizing, or stent type (Figure 3). In contrast, immediate post-stenting venous stenosis had a significant impact: patients with severe stenosis (≥50%) exhibited a marked reduction in primary patency, and this difference was statistically significant (Figure 3D).

Each graph shows the cumulative primary patency rates among patients with specific factors. Differences in the survival distribution were assessed using the log-rank test.

Stent diameter. Two-year primary patency according to stent diameter (≤12 mm vs. ≥4 mm). The ≥14 mm group showed numerically higher patency throughout follow-up; however, no statistically significant difference was observed between the two groups.

Stent oversizing. Two-year primary patency according to stent oversizing (≥20% vs. <20%). The ≥20% oversizing group showed a slightly higher patency rate during follow-up; however, the difference was not statistically significant.

Stent type. Two-year primary patency of vein-dedicated and non-venous-dedicated stents. The vein-dedicated stent group demonstrated a non-significant trend toward improved patency.

Post-stenting stenosis. Two-year primary patency according to the severity of residual stenosis on immediate post-stenting venography (absent, mild <50%, severe ≥50%). Severe residual stenosis was strongly associated with early reocclusion, resulting in a markedly lower patency than in the other groups (log-rank *p* < 0.001).

To identify the independent prognostic factors associated with 24-month primary patency, multivariate Cox proportional hazards regression analysis was performed, with stent diameter, stent oversizing, stent type, and in-stent stenosis as covariates (Table 3).

Among these variables, in-stent stenosis ≥50% emerged as the only statistically significant independent predictor of venous occlusion (HR = 4.625; 95% CI: 2.061–10.379; *p* < 0.001). This indicates that patients with significant residual stenosis had a 4.625–fold increased risk of reocclusion relative to those without stenosis. Stent diameter (HR = 1.025; 95% CI, 0.647–1.623; *p* = 0.917), stent oversizing (HR = 1.006; 95% CI, 0.980–1.032; *p* = 0.675), and stent type (HR = 0.274; 95% CI, 0.030–2.458; *p* = 0.247) were not significantly associated with the loss of primary patency.

## 4. Discussion

Achieving durable venous patency after thrombectomy remains a key therapeutic goal in patients with MTS-associated acute iliofemoral DVT, as the loss of patency is closely linked to recurrence and long-term morbidity. Endovascular treatment strategies, including catheter-directed thrombolysis and pharmacomechanical thrombectomy with subsequent stent placement, have become standard practice [5,6,7,8,9,10,11,12,13,14]; however, the factors that reliably predict long-term patency are still not fully established. Prior studies have largely emphasized the role of mechanical compression and the technical success of stent deployment. However, detailed venographic features observed immediately after thrombectomy, such as residual stenosis, luminal irregularity, and persistent collateral flow, have not been systematically evaluated as prognostic markers. A recent study by Oh et al. demonstrated that residual venous stenosis of ≥50% on completion venography after thrombectomy, with or without stenting, is a significant prognostic factor associated with impaired long-term patency in patients with symptomatic DVT and MTS [15]. Consequently, the prognostic implications of the stent-related variables remain unclear. To address this knowledge gap, the present study focused on identifying the venographic and procedural determinants associated with long-term patency following thrombectomy with iliac vein stenting in patients with MTS.

In this retrospective study of 50 patients with acute iliofemoral DVT due to MTS, we observed that iliac vein stenting led to excellent primary patency in most cases and identified residual stenosis ≥ 50% after stent placement as a critical risk factor for stent occlusion. These findings were consistent with those reported by Oh et al. [15]. Conversely, the stent diameter, oversizing, and type did not significantly influence patency in our study. These findings have important implications for the endovascular management of MTS-related DVT.

Our observed primary patency rates (88% at 1 year and 81.2% at 2 years) were consistent with the previously published outcomes of iliac vein stenting for MTS. Numerous studies have documented high patency and favorable clinical results in similar patient populations [5,6,8,9,10]. In our series, all occlusions occurred within the first year, and no new occlusions were observed thereafter in those with a longer follow-up period. This pattern suggests that if a stent remains patent during the initial healing period, long-term patency is likely to be maintained. This underscores the notion that early technical success and immediate postprocedural factors largely determine long-term outcomes. Our high patency rates reinforce the effectiveness of combining thrombectomy with iliac vein stenting to prevent recurrent thrombosis and occlusion in patients with iliofemoral DVT due to MTS, which is why current guidelines advocate an endovascular approach in this scenario [14].

The most notable finding of our analysis was a substantially increased risk of stent occlusion, which was associated with significant residual stenosis (>50%) after stent placement. Patients whose stented venous segments remained narrowed by more than 50% had an approximately 4.6-fold higher risk of reocclusion, underscoring the critical importance of achieving sufficient luminal restoration in the treatment of MTS. Residual stenosis likely reflects either incomplete stent expansion or an inadequately treated extension of the underlying lesion, both of which can maintain abnormal flow dynamics despite technically successful stent deployment. This observation aligns with a prior report [16] indicating that residual venous obstruction or an unresolved thrombotic burden is a major determinant of recurrent thrombosis. Our results specifically identified post-stenting residual narrowing as a modifiable and clinically actionable factor. Accordingly, surgeons should ensure that any significant stenosis is fully corrected during the index procedure. Strategies for optimizing luminal patency include aggressive balloon dilatation, selection of sufficiently large stents that cover the entire diseased or compressed segment, and routine use of intravascular US (IVUS) to assess the luminal dimensions accurately [3]. In addition, careful lesion preparation and confirmation of adequate stent expansion on completion venography or IVUS may help minimize residual stenosis. If significant narrowing persists, additional balloon dilatation or stent extension should be considered to achieve complete luminal restoration. Immediate correction rather than reliance on anticoagulation therapy alone may be warranted if substantial narrowing remains despite these measures. Overall, our findings suggest that leaving significant residual stenosis after stenting markedly increases the risk of early occlusion, even with appropriate post-procedural anticoagulation.

From a hemodynamic perspective, significant residual stenosis may disrupt normal laminar venous flow and create areas of flow disturbance and local stasis within the treated segment. Venous stasis is a key component of Virchow’s triad and plays a central role in the pathogenesis of venous thrombosis by promoting endothelial activation, platelet aggregation, and activation of the coagulation cascade [17]. These alterations in flow dynamics may facilitate thrombus formation and increase the risk of early stent thrombosis or recurrent occlusion despite technically successful stent deployment. This mechanism is particularly relevant in patients with May–Thurner syndrome, in whom chronic venous compression predisposes to abnormal flow patterns and a prothrombotic environment within the iliac vein [1].

In our study, stent diameter (10–12 mm vs. ≥14 mm) was not associated with differences in primary patency. Although larger stents may theoretically provide greater resistance to external compression, we did not observe a difference in occlusion rates within the diameter ranges used. This finding is consistent with previous reports demonstrating favorable outcomes after iliac vein stenting for MTS without a significant association between stent diameter and patency [13,18]. One possible explanation is that, once adequate luminal expansion is achieved and the stenotic segment is fully treated, the absolute stent diameter may be less important than the completeness of lesion correction. In our cohort, all stents were at least 10 mm in diameter, which is generally sufficient for the iliac venous system [12,19]. Many experts advocate using the largest feasible stent (often 14–16 mm for the common iliac vein) to ensure the stent can oppose the vein wall and resist compression [20,21]. Our data suggest that using 10–12 mm stents, when appropriately selected for smaller veins or distal extensions, did not result in increased occlusions, provided that no significant residual stenosis remained. Therefore, achieving adequate stent expansion and elimination of significant residual stenosis may be more critical determinants of long-term patency than the nominal stent diameter.

In our study, stent oversizing was not associated with primary patency. This suggests that the degree of oversizing itself may have a limited influence on long-term stent durability in MTS-related iliac vein obstruction. Instead, our findings indicate that eliminating significant residual stenosis after stent deployment is more important than the exact degree of oversizing. Although stent sizing should generally correspond to the diameter of the adjacent normal venous segment, the final clinical outcome appears to depend more on achieving adequate luminal restoration than on the precise percentage of oversizing.

Similarly, we observed no difference in patency between dedicated venous stents and nondedicated stents. Although dedicated venous stents offer design advantages such as minimal foreshortening and optimized radial force [11,21], both stent types demonstrated satisfactory patency in our cohort. These findings are consistent with previous reports showing comparable outcomes between dedicated venous stents and traditional stent constructs in the treatment of iliofemoral venous obstruction [21]. This may indicate that procedural factors, including accurate positioning and complete expansion of the stent, play a greater role in determining patency than the specific stent design itself.

Our study had some limitations. This study was retrospective and single-center with a relatively small sample size. This limits the statistical power and ability to perform a robust multivariate analysis. A larger cohort would help refine the exact magnitude of the risk. In addition, the relatively small number of patients within certain subgroups, particularly those treated with dedicated venous stents, may have further limited the statistical power to detect meaningful differences between stent types. Therefore, the absence of a significant association between stent type and patency in our analysis should be interpreted with caution, as a potential Type II error cannot be excluded. Second, there may also be selection bias in those who received the stent type or size, as the device choice was not randomized. For instance, operators may have used larger stents in more severe cases or larger patients, and such nuances are difficult to adjust for fully. We also focused on stent-related technical factors and did not systematically analyze patient-specific factors, such as thrombophilia, clot burden, or adjunctive lytic therapy vs. no lysis. Third, the follow-up duration, while capturing short- and mid-term outcomes, varied among patients; however, long-term differences in stent performance (such as 5- to 10-year patency or late complications) were beyond the scope of this study. Finally, our definition of residual stenosis was based on subjective venographic assessment. In contemporary practice, IVUS or cone-beam CT would provide a more objective measurement of the percent stenosis or minimal luminal area; however, our data were limited by what was recorded in the charts. Despite these limitations, this study provides valuable insights as one of the few studies focusing on the technical risk factors for stent patency in acute DVT in the MTS context. Real-world data add to the growing evidence that endovascular treatment of MTS is effective and highlight a key modifiable factor (residual lesion) that interventionists should address.

## 5. Conclusions

Thrombectomy with iliac vein stenting for MTS-related DVT achieved high primary patency, with occlusions occurring only in the early period. Residual stenosis ≥50% after stent placement was the only factor associated with stent failure, whereas stent diameter, oversizing, and stent type showed no impact. Eliminating significant residual stenosis during the index procedure is essential for achieving long-term patency. Further prospective studies with larger patient populations are warranted to validate these findings and to better define optimal procedural strategies for minimizing residual stenosis.

## Figures and Tables

**Table 1:** Baseline clinical and procedural characteristics of the study population.

	Total (*n* = 50)
Mean age (years)	68 ± 15.7
Sex, *n* (%)	
Male	10 (20)
Female	40 (80)
Symptom onset to treatment (days)	5.5 ± 3.6
Stent diameter (mm)	13.5 ± 1.7
10	2 (4)
12	18 (36)
14	21 (42)
16	8 (16)
18	1 (2)
Stent type	
Vein-dedicated stent	13 (26)
Non-vein dedicated stent	37 (74)

**Table 2:** Comparison of stent-related and post-stenting venographic characteristics between patent and occluded groups.

		Patent (*n* = 41)	Occluded (*n* = 9)	*p* Value
Stent diameter	≤12 mm	15	5	0.277
	≥14 mm	26	4	
Stent oversizing	≥20%	30	5	0.402
	<20%	11	4	
Stent type	Vein dedicated	12	1	0.269
	Non-vein dedicated	29	8	
Post-stenting stenosis	Absent	34	3	<0.001
	<50% (mild)	0	5	
	≥50% (severe)	7	1	
Persistent collaterals	Absent	41	9	N/A
	Present	0	0	

**Table 3:** Multivariate Cox Proportional Hazards Regression Analysis for a 24-month Primary Patency.

	Hazard Ratio	95% Confidence Interval	*p* Value
Stent diameter	1.025	0.647–1.623	0.917
Stent oversizing	1.006	0.980–1.032	0.675
Stent type	0.274	0.030–2.458	0.247
In-stent stenosis	4.625	2.061–10.379	<0.001

## Data Availability

Data generated or analyzed during the study are available from the corresponding author by request. The data are not publicly available due to privacy, legal reasons.

## Abbreviations

The following abbreviations are used in this manuscript:

MTS	May–Thurner syndrome
DVT	Deep vein thrombosis
PTS	Post-thrombotic syndrome
US	Ultrasound
USA	United States of America
CT	Computed tomography
CI	Confidence interval
HR	Hazard ratio
IVUS	Intravascular ultrasound
